# Correlative Conventional and Super-resolution Photoactivated Localization Microscopy (PALM) Imaging to Characterize Chromatin Structure and Dynamics in Live Mammalian Cells

**DOI:** 10.21769/BioProtoc.4850

**Published:** 2023-10-20

**Authors:** Dushyant Mehra, Elias M. Puchner

**Affiliations:** 1 School of Physics and Astronomy, University of Minnesota, Twin Cities, MN, USA; 2 Department of Physiology and Biomedical Engineering, Mayo Clinic, Rochester, MN, USA

**Keywords:** Photoactivated localization microscopy, PALM, Single-molecule tracking, Nanoscale, Chromatin structure and dynamics, Live-cell chromatin imaging, CRISPR/dCas9 DNA-labeling, MS2 gRNA, Live-cell super-resolution imaging

## Abstract

A fundamental understanding of gene regulation requires a quantitative characterization of the spatial organization and dynamics of chromatin. The advent of fluorescence super-resolution microscopy techniques such as photoactivated localization microscopy (PALM) presented a breakthrough to visualize structural features with a resolution of ~20 nm in fixed cells. However, until recently the long acquisition time of super-resolution images prevented high-resolution measurements in living cells due to spreading of localizations caused by chromatin motion. Here, we present a step-by step protocol for our recently developed approach for correlatively imaging telomeres with conventional fluorescence and PALM, in order to obtain time-averaged super-resolution images and dynamic parameters in living cells. First, individual single molecule localizations are assigned to a locus as it moves, allowing to discriminate between bound and unbound dCas9 molecules, whose mobilities overlap. By subtracting the telomere trajectory from the localization of bound molecules, the motion blurring is then corrected, and high-resolution structural characterizations can be made. These structural parameters can also be related to local chromatin motion or larger scale domain movement. This protocol therefore improves the ability to analyze the mobility and time-averaged nanoscopic structure of locus-specific chromatin with single-molecule sensitivity.

## Background

Gene expression is thought to be regulated by the spatiotemporal organization of chromatin from the smallest length scale of individual nucleosomes (~10 nm) up to ~100 nm [1,2]. Even larger tertiary structures such as enhancer promoter contacts or topologically associated domains may exist that regulate gene expression [1,3]. The correlated movement between small chromatin structures and the large chromatin domains they are part of has been suggested as an important feature of nuclear phase condensates [4–6]. To understand these effects of chromatin structure and dynamics on gene regulation, imaging techniques are required that can characterize both the nanoscopic structure and the dynamics of chromatin in the larger context of chromatin domains they reside in.

Recently, two main breakthroughs facilitated research advances in this field: CRISPR/dCas9-based fluorescence labeling methods, to image specific sequences of chromatin, and super-resolution microscopy techniques such as photoactivated localization microscopy (PALM). By using programmable guide RNAs (gRNA), fluorophores can be targeted via CRISPR/dCas9 to specific sequences in the genome. Tens of fluorescent probes are required to be bound to a locus of interest to create a signal that is distinguishable from the background fluorescence of all other freely diffusing and searching probes [7]. To amplify the fluorescence signal of bound probes, repetitive RNA aptamers such as MS2 sequences have been attached to gRNAs that facilitate binding of multiple fluorophores. These labeling strategies facilitated conventional fluorescence timelapse imaging and yielded valuable insights into the slow and long-term dynamics of entire loci [7–9]. However, due to the optical diffraction limit, the structural characterization of smaller chromatin structures below ~250 nm has not been possible with conventional fluorescence microscopy. The development of PALM [10–12] enabled the tracking of single molecules in living cells (also referred to as single-particle tracking PALM, sptPALM) [13,14] and the acquisition of images of intracellular structures in fixed cells with ~20 nm resolution [15]. In PALM imaging, the spatiotemporal overlap of individual fluorophores is avoided by sparsely activating photoactivatable or photoswitchable fluorophores. In this way, the precise locations of individual fluorophores can be determined by Gaussian fitting of their intensity profile. By photoactivating and localizing many or all fluorophores over time, enough localizations are obtained to resolve structures below the optical diffraction limit in fixed cells or to link molecular trajectories to characterize their diffusion in live cells. CRISPR/dCas9-based DNA labeling has recently been applied in PALM to obtain structural information of chromatin compaction or condensation in chemically fixed cells or to characterize the dynamics of chromatin in live eukaryotic and prokaryotic cells [16]. However, until recently, it was not possible to simultaneously obtain such structural and dynamic information in living cells due to the motion of DNA during the long PALM data acquisition time. This motion spreads out the localizations of bound fluorophores along the trajectory of a locus and thus results in motion blurring.

Here, we present a protocol for our recently developed correlative conventional fluorescence and PALM imaging approach in living cells that overcomes these challenges [17]. This approach is based on labeling an intracellular structure or locus with a conventional fluorophore to track its location and motion during the entire PALM data acquisition time. Each structure or locus is, in addition, labeled with a spectrally distinct PALM-compatible fluorophore to record the single molecule localizations. The trajectory of the locus determined from the conventional fluorescence signal is then subtracted from the coordinates of its single molecule localizations to correct for motion blurring. As a result, high-resolution structural parameters such as the time-averaged size or the density of bound probes can be quantified to obtain new insights into the compaction of chromatin. In addition, the dynamics of the relative single molecule rearrangement or the motion of the entire locus can be related to its structural parameters, which has not been possible with existing techniques.

We demonstrate correlative conventional and PALM imaging using the well-characterized telomere sequences as a model system labeled via dCas9 and the MS2 coat proteins (MCP) that bind to a modified telomere-targeting gRNA scaffold. However, this imaging approach can be extended to single loci or other intracellular structures that can be labeled with a sufficient number of conventional and PALM-compatible fluorophores to create signals above background. Importantly, our presented data acquisition and analysis pipeline is a primary step to any downstream analysis to quantify structural or dynamic parameters. For instance, we demonstrate that determining the location and mobility of a locus relative to the single dCas9/MCP fluorophores classifies them more reliably as bound to a locus. The relative motion of single molecules compared to the entire locus furthermore reveals how small-scale chromatin rearrangements occur within the larger-scale chromatin movements. We also relate the compaction of telomeres to the local and global chromatin mobility to yield new insights. This protocol demonstrates that correlative conventional fluorescence and PALM imaging accurately identifies Cas9 molecules bound to a locus and yields quantitative dynamic and time-averaged structural information about specific genomic loci at the nanoscale in living cells. The versatility of this protocol makes it applicable to other organelles and enables other existing or future downstream analysis techniques to extract and correlate high-resolution structural features with dynamic parameters.

## Materials and reagents

1. Lab-Tek No. 1.5 8-well plates (Fisher Scientific, catalog number: 12-565-8)
2. Lipofectamine 3000 and p300 reagent (Invitrogen, catalog number: L3000001)
3. Opti-MEM media (Thermo Fisher, catalog number: 31985070)
4. MCP-HaloTag plasmid (Addgene, catalog number: 121937)
5. dCas9-GFP plasmid (Addgene, catalog number: 51023)
6. 2xMS2 gRNA plasmid (Addgene, catalog number: 75389)
7. PA-JF646 (Luke Lavis Lab, HHMI Janelia)
8. GIST-T1 Cells (gastrointestinal stromal tumor cells) (Cosmo Bio, catalog number: PMC-GIST01C)
*Note: We used this cell line as it is hypothesized that this cancer phenotype is impacted by changes in chromatin structure and dynamics [18]. Furthermore, living GIST-T1 cells can be imaged for long periods of time and exhibit minimal auto-fluorescence and cell death.*
9. T25 tissue culture flask (Thermo Fisher, catalog number: 156340)
10. Phenol-red free trypsin EDTA (Gibco, catalog number: 15400054)
11. Fetal bovine serum (FBS) (Gibco, catalog number: 10437028)
12. Fluorobrite DMEM (Gibco, catalog number: A1896701)
13. Penicillin/Streptomycin (Gibco, catalog number: 15140122)
14. L-Glutamine (Gibco, catalog number: 25030-081)
15. TetraSpeck microspheres (Invitrogen, catalog number: T7279)
16. 1.7 mL Eppendorf tubes (catalog number: 0030123611)
17. 37 °C 5% CO_2_ incubator (Thermo Fisher, catalog number: 3110 or similar)
18. DI water (e.g., Thermo Fisher, catalog number: 751-610 or purified in house)
19. Distilled phosphate buffered saline (PBS) (Gibco, catalog number: 14040133)

**Solutions**

1. Fluorobrite media (see Recipes)
2. Serum-diluted Fluorobrite media (see Recipes)
3. DNA/lipid mixture (see Recipes)

**Recipes**

1. **Fluorobrite media**
  10% FBS, 4 mM L-Glutamine, 1% penicillin/streptomycin, Fluorobrite DMEM. For 50 mL of media, add 5 mL of FBS, 500 μL of penicillin/streptomycin solution, 500 μL of L-Glutamine, and 44 mL of Fluorobrite DMEM.
2. **Serum-diluted Fluorobrite media**
  1% FBS, 4 mM L-Glutamine, 1% penicillin/streptomycin, Fluorobrite DMEM solution. For 50 mL of media, add 500 μL of FBS, 500 μL of penicillin/streptomycin solution, 500 μL of L-Glutamine, and 48.5 mL of Fluorobrite DMEM.
3. **DNA/lipid mixture**
  Mix 200 ng of telomere 2x MS2 gRNA along with 50 ng of MCP-HaloTag and 50 ng of dCas9-GFP plasmids with 10 μL of Opti-MEM, 1 μL of Lipofectamine 3000 reagent, and 0.5 μL of p300 reagent in a 1.7 mL Eppendorf tube in sterile cell culture environment.
*Note: Telomere gRNA sequence was obtained from [7], and protocols from [8,19], and [9] were used to clone telomere gRNA sequence into 2xMS2 plasmid.*

## Equipment

1. Four OBIS lasers emitting 100 mW at 405 nm (Coherent, catalog number: 1178754), 50 mW at 488 nm (Coherent, catalog number: 1178764), 100 mW at 561 nm (Coherent, catalog number: 1253302) and 100 mW at 640 nm (Coherent, catalog number: 1178790)
2. Beam expander (Thor Labs, catalog number: GBE02-A)
3. Assorted lenses and mirrors (Thor Labs)
4. Inverted microscope (Eclipse Ti-E) equipped with a perfect focus system (Nikon, catalog number: MEA53100)
5. CFI 100× 1.49 NA oil immersion objective (Nikon, catalog number: MRD01991)
6. iXon 897 Ultra DU-897U EMCCD camera (Andor, catalog number: 77026047)
7. Quad band dichoric mirror (Chroma, catalog number: ZT405/488/561/640rpc)
8. Bandpass filters for the green (Chroma, catalog number: ET525/50), red (Chroma, catalog number: ET595/50), and far-red channel (Semrock, catalog number: FF731/137)
9. Dichroic long pass beam splitter for red/green channel experiments (Chroma, catalog number: T562lpxr BS)
10. Dichroic long pass beam splitter for far red/green channel experiments (Semrock, catalog number: FF652-Di01)
11. Motorized flat top stage for inverted microscope (ProScan II, catalog number: 77011328)
12. Heating insert P for Lab-TekTM S1 and temperature controller (Pecon, catalog number: 411860-9025-000 and 411860-9005-000)
13. Computer for microscope control and data acquisition (e.g., Dell, model: Optiplex 9020 Mini-Tower, Intel Core i7-4790 CPU @3.60GHz 4 cores, 16 GB RAM, 3.64 TB drive)
14. Computer for data analysis (e.g., Dell, model: PowerEdge T440, Intel Xeon Silver 4216 2.1G, 16C/32T, 9.6 GT/s, 22 M Cache, 16 GB RDIMM, 3,200 MT/s, Dual Rank, 8 TB 7.2K RPM SATA 6Gbps 512e 3.5 in Hot-plug Hard Drive)

## Software

1. MATLAB 2018b
2. Insight3 Localization Software (Huang lab, UCSF or Zhuang lab, Harvard) or equivalent
3. Storm Control Software (https://github.com/ZhuangLab/storm-control) or equivalent
4. MATLAB-based trace linking and trace analysis (https://osf.io/6n4ej/)
5. MATLAB-based motion correction and trace separation code (https://osf.io/6n4ej/)
6. Python-based channel transformation code (https://osf.io/6n4ej/)

## Procedure

- **GIST-T1 cell culture, seeding, and plasmid transfections**
  1. Seed GIST-T1 cells in 5 mL of Fluorobrite media at a density of 33% (~400,000–600,000 cells) in a T25 tissue culture flask.
*Note: Fluorobrite media avoids fluorescence from phenol red.*
  2. Culture cells for ~1–2 days up to a density of 75%–80% in a humidified incubator at 37 °C and 5% CO_2_. Split cells by aspirating media from the flask in a cell culture hood and washing cells with 37 °C PBS twice.
  3. After removing PBS, add 0.5 mL of phenol-red free trypsin EDTA to the flask and make sure the entire surface area of the flask is covered.
  4. Then, place flask in incubator for 2–3 min to allow cells to lift from flask. Verify that cells have lifted.
  5. Add 2 mL of Fluorobrite media to trypsinized cells to neutralize trypsin and add cells at density mentioned in step A1 with 5 mL of Fluorobrite media or proceed with step A6.
  6. Seed 50 μL of trypsinized cells in 8-well plates at a density of 50,000 cells/mL two days prior to imaging. Add 400 μL of media to each well after plating cells, and culture as in step A1.
*Note: Cell concentration was measured using a hemocytometer, and Fluorobrite media was used to dilute cells to find appropriate concentration.*
  7. Approximately 15–17 h before imaging, mix 200 ng of telomere 2xMS2 gRNA plasmid to generate gRNA along with 50 ng of MCP-HaloTag and 50 ng of dCas9-GFP plasmids with 10 μL of Opti-MEM, 1 μL of Lipofectamine 3000 reagent, and 0.5 μL of p300 reagent in a 1.7 mL Eppendorf tube and incubate the DNA/lipid mixture for 15 min at room temperature in a sterile cell culture environment.
  8. During incubation of step A7, aspirate media from GIST-T1 cells in a well plate and wash cells twice with 300 μL of Fluorobrite media heated to 37 °C. Then, add 100 μL of Fluorobrite media supplemented with 4 mM L-Glutamine, 1% FBS, 1% penicillin/streptomycin, and 200 μL of Opti-MEM. Then, place cells in an incubator at 37 °C with 5% CO_2_ for 15 min.
  9. After 15 min of incubation in step A8, remove cells from incubator, place in cell culture hood, and add all of the DNA/lipid mixture from the Eppendorf tube to cells in each well already supplemented with serum-diluted media by pipetting dropwise. If too much force is used, DNA/lipid complex may disassociate.
  10. Place wells with transfecting cells in the incubator as in step A2 for 15–17 h.
  11. Remove media from wells and wash cells twice with serum-diluted Fluorobrite media. Add 300 μL of serum-diluted Fluorobrite media with 100 nM of PA-JF646 dye and incubate for 15 min in a 37 °C incubator with 5% CO_2_.
*Note: PA-JF646 is a far-red fluorescent dye that is photoactivated by 405 nm light and contains a ligand that attaches to HaloTag [20,21]. This dye has a higher photon budget and longer on-times compared to other photoswitchable proteins, which results in improved detection, localization precision, and longer single molecule trajectories. This improves trace mobility analysis and diffusion coefficient estimation. This dye isn’t fluorogenic and does fluoresce when not bound to HaloTag, which is why multiple rounds of washing are required before imaging.*
  12. After incubation, wash cells three times with Fluorobrite media and place in an incubator at 37 °C with 5% CO_2_ for an additional 30 min.
  13. Repeat step A12 three additional times prior to imaging to remove unbound PA-JF646 dye that is still able to fluoresce.
  14. Keep samples in an incubator at 37 °C with 5% CO_2_ until imaging.
- **Calibration and imaging experiments**
*Note: All experiments presented here were performed with a custom-built microscope, as recently described [22]; however, our protocol is applicable to data recorded with any microscope capable of simultaneous PALM and conventional fluorescence imaging in the respective channels. In short, a Nikon inverted microscope (Eclipse Ti-E) is equipped with a perfect focus system and an Andor iXon 897 Ultra DU-897U electron multiplying charge coupled detector (EMCCD), cooled to -70 °C and set to an amplifying gain of 30. The four 100 mW excitation lasers (405, 488, 561, and 640 nm OBIS-CW, Coherent Optics) are aligned, expanded, and focused into the back focal plane of the objective (Nikon CFI 100× 1.49 NA oil immersion) using various dichroic mirrors, beam expanders, and lenses. A quad band dichoric mirror (ZT405/488/561/640rpc; Chroma) separates fluorescence emission from excitation light. Fluorescence emission is further split into the far red and green signal using a dichroic long pass beam splitter (FF652-Di01; Semrock) and band pass filters FF731/137 (Semrock) for the far-red channel and ET525/50 (Chroma) for the green channel. Laser intensity modulation and shutter sequences are synchronized with the camera and controlled digitally with a NI-DAQ board.*
  1. Mount microscope No. 1 cover glass or 8-well plates with 10 μL of TetraSpeck microspheres diluted 1:100 in DI water on microscope stage.
  2. Excite microspheres separately using 640 and 488 nm excitation with approximately 1.75 mW power (power density ~100 W/cm^2^ in sample plane). Record a 100-frame movie with approximately 10 sparsely distributed microspheres over the entire field of view without moving the sample and use the first 50 frames to record 640 nm excited microspheres and the last 50 frames to record 488 nm excited microspheres.
  3. Move the same microspheres to different positions in the camera to sample the entire field of view and repeat steps B2 and B3 at least five times. You can also image different microspheres placed in different regions across the field of view.
*Note: The number of microspheres used depends on the field of view of the camera. Our field of view was 256 × 256 pixels with a pixel size of 160 nm. You need at least 50 microspheres to create an accurate transformation with sub 20 nm registration error (see Figure 1). More microspheres or more images with microspheres shifted to different locations are necessary if your field of view is larger. Microsphere imaging can also be done after cellular imaging but should be done either before or after every imaging session, since registration parameters can change from session to session. This data will be used later to transform localizations from 640 nm channel to the 488 nm channel.*
    **Figure 1.**
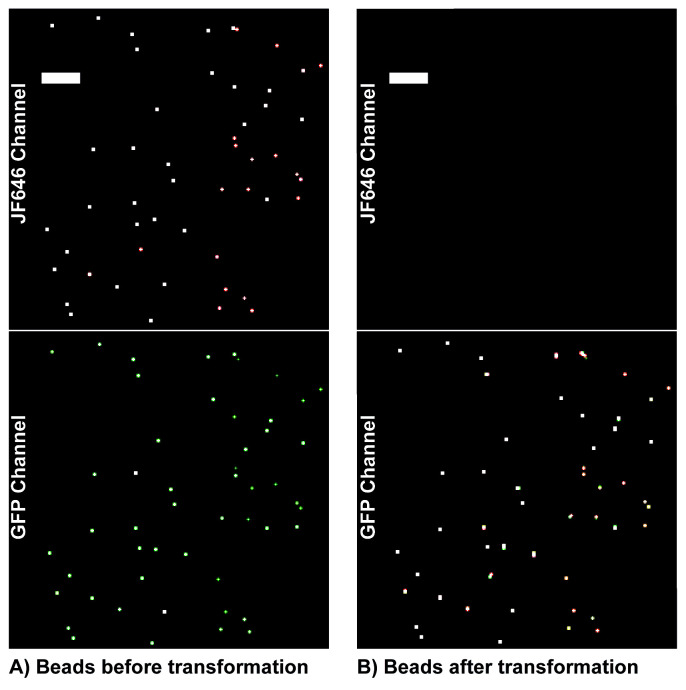
Example microsphere localization rendering. Example rendering of microsphere localizations across seven movies depicting the number and density of microspheres required to obtain an accurate transformation across the field of view. (A) Microspheres imaged in each channel to calculate the transformation matrix. (B) The transformation matrix is applied to transform bead localizations from the Jf646 channel to the GFP channel. This transformation matrix is then applied to single molecule localizations. Scale bars: 5 µm.
  4. Remove microsphere sample and mount 8-well plate with transfected cells on microscope stage. Make sure the stage is heated to 37 °C and the CO_2_ incubator on microscope stage reads 5%.
  5. Using the Storm Control Software (or equivalent for a different microscope system), set up a 10-frame laser shutter sequence at 20 Hz with the first frame having 488 nm excitation for GFP imaging, the second having brightfield LED and 405 nm photoactivation, and frames 3–10 having 640 nm excitation for PALM imaging.
*Note: The diffusion coefficient distributions of MCP-HaloTag with and without telomere gRNA taken at 20 Hz are statistically similar to the ones obtained at 60 Hz. While faster PALM imaging speeds in principle reduce motion blurring, the required higher excitation power reduces the length of single molecule traces, and thus lowers the precision for characterizing their motion. In our experience, a 20 Hz frame rate has been a good compromise between imaging speed, localization precision in each frame, and single molecule trace lengths.*
  6. Set the 488 nm laser power to 1.75 mW (power density ~100 W/cm^2 ^at sample plane) and the 640 nm to 17.5 mW (~1 kW/cm^2^). The 405 nm intensity will be adjusted to 1–251 μW (power density of ~0.06–15 W/cm^2^) during the experiment for constant photoactivation rates.
  7. Turn on the 488 nm laser to visually identify cells with telomere puncta in cell nucleus prior to single molecule imaging. Keep 488 nm exposure to a minimum to reduce bleaching. Image telomere puncta conventionally using the 488 nm laser for at least 200 frames at 20 Hz for interpolation error analysis.
*Note: Cells transfected with all three plasmids should show clear telomere puncta. Since cells are transiently transfected, some cells express all transfected plasmids while others do not. Look at cells that do not have gRNA but have MCP-HaloTag+PA-JF646 dye and dCas9-GFP expressed, and cells stained with only PA-JF646 dye and no transfected plasmids to characterize single molecule and conventional fluorescence background (see Figure 2).*
    **Figure 2.**
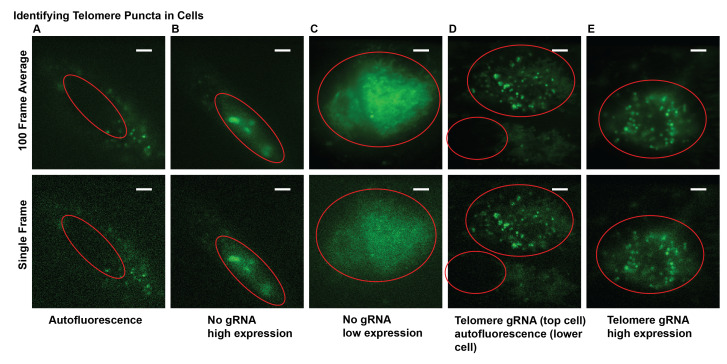
Examples of fluorescence images from transfected cells suitable and unsuitable for experiments. (A) Example of cellular autofluorescence. (B) and (C) Examples of cells expressing dCas9-GFP at various levels but without distinct telomere puncta due to the lack of gRNA. This can also occur with gRNA added. (D) and (E) Examples of telomere puncta at various expression levels suitable for imaging and analysis. Nuclei are marked with red line. Scale bars: 5 μm.
  8. Once cells with telomere puncta are identified, begin 10-frame shutter and acquisition sequence. Start with 405 nm laser power of 1 μW (power density of ~0.06 W/cm^2^) and slowly increase at a rate of 5–10 μW every 1,000 frames until approximately 250 μW (power density of ~0.015 W/cm^2^) to ensure a sparse and consistent photoactivation rate (see Figure 3).
    **Figure 3.**
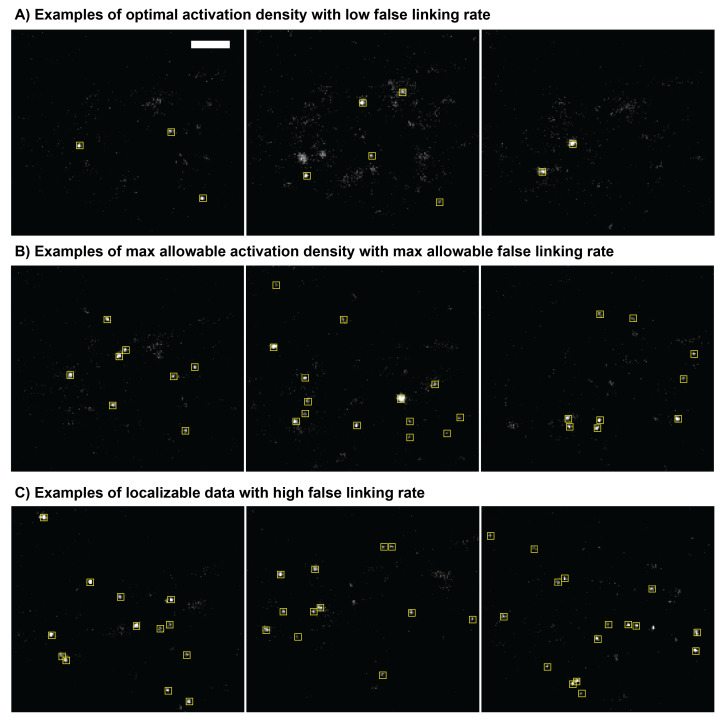
Optimal photoactivation density. (A) Single frame of an optimal photoactivation density for single molecule tracking with low false linking rate. Yellow boxes are identified localizations from single-molecule emission events. (B) Example images of the maximal allowable localization density for single molecule tracking with acceptable false linking. (C) Examples of images with high-localization density that would result in a high false linking rate. False linking rates for example images are quantified in Figure 5. Scale bar: 5 μm.
  9. Record data for 15,000–30,000 frames and stop sequence when cells start to change morphology indicating a decline of cell health (see Figure 4).
*Note: Healthy GIST-T1 cells never change their morphology up to 15,000 frames. Only analyze and use frames up to 5,000 frames before morphology changes are detected, to exclude potential phototoxic effects prior to morphological changes. If cells start to change morphology sooner, cells are not healthy enough to begin with for imaging experiments (see Figure 4).*
    **Figure 4.**
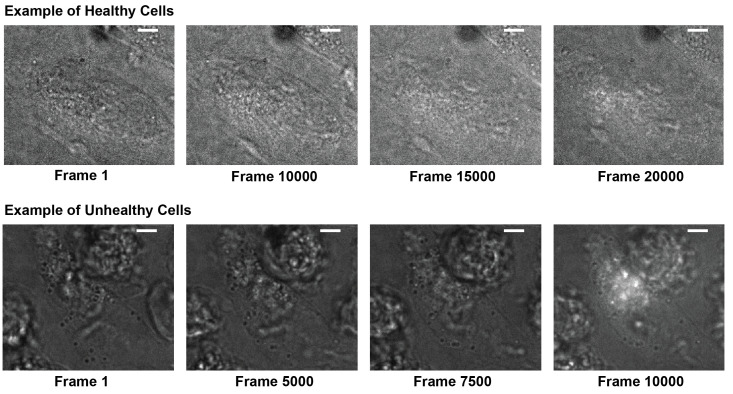
Cell morphology and health. LED + 405 nm photoactivation frames of healthy GIST-T1 cells (top) typically show retained morphology and size for at least 15,000 frames and show minimal autofluorescence upon 405 nm activation. Unhealthy cells (bottom) change morphology and become round within several thousand frames and show significant autofluorescence in response to 405 nm activation. Healthy cells also show much less contrast in the LED frame due to a more homogenous refractive index. Scale bars: 5 μm.
  10. Repeat steps B5–B9 to obtain more statistics from different cells. Typically, a number on the order of 10 cells is considered satisfactory.

## Data analysis

- **Single molecule localization**
  1. Use Insight3 localization software or a similar single molecule localization microscopy software to identify single molecule localizations and to fit them with 2D elliptical Gaussians with the following parameters: 7 × 7 pixel ROI, widths between 250 and 700 nm, and minimum intensity of 100 photons. The x and y coordinate of each localization, along with the intensity, width, background, frame number, and other parameters are also stored in the single molecule list and outputted as a .txt file for further analysis.
*Note: Other localization detection software, such as Thunderstorm or SMAP, can also be used with similar parameters [23,24]. Make sure to export localization list as .txt file and to include PSF widths.*
  2. Import molecule list into provided MATLAB analysis package or equivalent single particle tracking software. If using Insight3 for localization detection, insert file path into LoadMoleculeLIst.m and execute function to generate a molecule list structure that will be used for subsequent analysis steps.
  3. Use Thompson resolution formula or more accurate and updated version [25,26] to calculate localization precision of single molecule localizations. If using Insight3 to localize images, input molecule list into function ThompsonResolution.m to calculate localization resolution for each localization.
*Note: The localization precision is provided in most localization software packages, such as Thunderstorm or SMAP along with localizations.*
- **Single molecule trace linking and trace analysis**
  1. To perform trace-linking error analysis, execute the MATLAB function spatiotemporal_cc.m and provide the molecule list structure and desired frame range for the analysis. Use this function to measure pairwise distance of localizations in same frame (see Figure 5).
*Note: This function normalizes the number of pairwise distances in each bin by the area and number of frame pairs. This modified pair correlation function quantifies the number of molecules found around each localization in the same frame. The number of localizations at your desired linking distance in the same frame will give you the estimate for the false linking rate.*
    **Figure 5.**
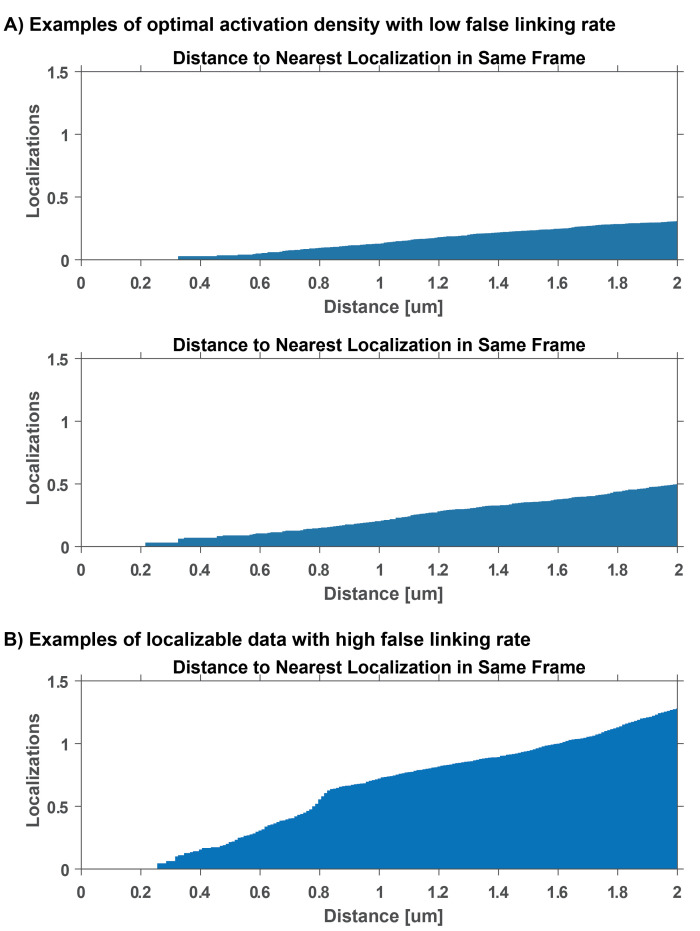
Localization density and false linking rate. False linking rate calculations for examples in Figure 3. These plots depict the average number of localizations as a function of distance away from a localization in the same frame. The false linking rate can be estimated by obtaining the number of localizations at a specific trace linking distance. (A) Optimal false linking rates for 0.48 μm linking distance is between 0.01 and 0.05. (B) A linking error at 0.48 μm of approximately 0.1 is considered a high false linking rate and will lead to unreliable mobility measurements.
  2. Exclude data from further analysis if localization density is too high and if the false linking rate is above 0.1. If false linking rate is too high throughout the duration of the experiment, record data with lower 405 nm photoactivation power in step B8.
*Note: If you want to allow dark frames for linking a single molecule trace, incorporate the number of dark frames in the pair correlation metrics. For example, if you use one dark frame, then you need to calculate pairwise distances between every two frames to calculate false linking error.*
  3. Based on results from step 1, link localizations that are within the determined distance threshold. In the datasets presented here, a 0.48 μm linking distance was used. Only traces with a minimum of four localizations in consecutive frames are used for cross correlation and single trace fitting analysis. Input the data structure from LoadMoleculeList.m, linking distance (in μm), and number of dark frames into the MakeTraces.m function to link molecules across successive frames into traces. Any equivalent trace linking analysis package can also be used.
  4. Calculate mean squared displacements (MSD) for each time step in all single molecule traces and average MSDs for each timestep of a single trace to obtain a time-averaged mean squared displacement (TAMSD) vs. time plot. If using the provided MATLAB code, input the output data structure from MakeTraces.m into function DiffusionDisplacment.m and execute DiffusionDisplacement.m.
  5. Fit each TAMSD vs. time to the 2D diffusion equation <r^2^> = 4DΔt + 2σ^2^, where D is the diffusion coefficient, Δt is the time step, r^2^ is the TAMSD, and σ is the localization precision (see Figure 6). Only use trajectories and diffusion coefficients from fits with a coefficient of determination value (R^2^) of 0.7 or above and a diffusion coefficient above zero. If using the provided MATLAB code, execute DiffusionCoefficientAnalysis.m function, which calculates the diffusion coefficient and R^2^ value for each input TAMSD calculated from DiffusionDisplacement.m.
  **Figure 6.**
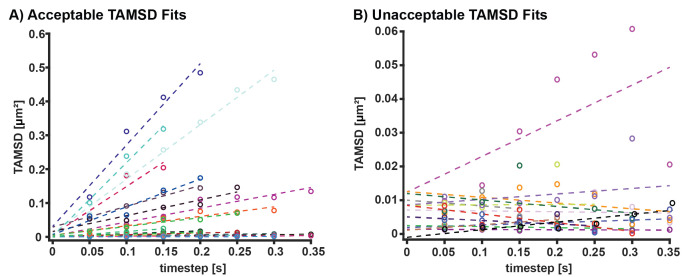
Time-averaged mean squared displacement (TAMSD) fitting. (A) Examples of acceptable TAMSD fits. These fits have R^2^ values of above 0.7, positive slopes, and y intercepts above 0. The y intercept can be used to calculate localization precision. (B) Examples of excluded TAMSD fits. These fits have either R^2^ values of below 0.7, negative slopes, or y intercepts at or below 0.
- **GFP cluster identification and trace linking analysis**
  1. Obtain GFP localizations by fitting GFP clusters with 2D Gaussians as in step A1 from Data analysis with the following parameters: 11 × 11 pixel ROI, widths between 250 and 7,000 nm, and a minimum of 200 photons. X and y coordinates of the GFP localization, along with the intensity, width, background, frame number, and other parameters will again be stored in the molecule list.
*Note: Even though the width range is large, make sure widths from the same telomere do not deviate significantly. If the GFP localization widths from the same telomere deviate by more than ~200 nm, it indicates that the telomere is moving along z too much and cannot be used for further analysis. If using other localization software such as SMAP or Thunderstorm, make sure PSF widths are included in the output molecule list.*
  2. To link telomere GFP localizations that are within 0.48 μm of each other in consecutive conventional imaging frames (every 10 frames), repeat steps B3–B5 with the GFP localization list.
  3. Only use traces with a minimum of five localizations for downstream analysis. The widths of consecutive localizations in a trace should be within 200 nm to be included in downstream analysis. If using steps B3–B5, this will automatically be done in the motioncorrection.m function.
*Note: An axial microsphere calibration [22] showed that PSFs width deviations of 200 nm corresponded to axial deviations of 450 nm, which is similar to the lateral trace linking threshold. In our analysis, we found that traces with below five localizations had insufficient single molecule localizations for downstream motion correction analysis.*
  4. Perform a linear interpolation between the x and y coordinates of GFP localizations in consecutive conventional image frames (frame 1 and frame 10) to obtain interpolated GFP coordinates during the frames that contained single molecule localizations. If using steps B3–B5, this will automatically be done in the motioncorrection.m function.
  5. To estimate the upper limit of the interpolation error, analyze the data of step B7 of the calibration and imaging experiments in the same way as steps B1–B4 of Data analysis. Compare interpolated positions between frames nine frames apart to the actual position of the telomere.
*Note: The median interpolation error of the presented data is 45 ± 10 nm and the mean interpolation error is constant up to 20 frames (Supplementary Figure 4 in reference [17]).*
  6. Calculate TAMSD and diffusion coefficients using the same procedure described in step B5 of Data analysis. Only use the first four time steps for fitting analysis to exclude nonlinear portions of the TAMSD that occur at later time steps. In this way, non-Brownian diffusion is approximated and sub-diffusive behavior at long times is not given too much weight in the fit.
- **Motion correction of single molecule localizations**
  1. Localize microspheres from calibration images recorded in steps B1–B3 in both channels using Insight3 localization software or equivalent with parameters used to localize single molecules in step A1 of Data analysis.
  2. Fit the positions of the microspheres in both channels to a third order polynomial function to extract the coordinate transformation matrix between the two channels. If using the provided procedure, input the two molecule lists and execute the python polynomial transformation code bead_calibration.py [27,28].
  3. Apply transformation to top channel to superimpose localizations from 640 nm channel to bottom channel. If using Insight3 localization software, go to STORM math in the STORM panel, click the custom math function, and copy the output transformation equations from the python transformation code into the *storm math* text box. Then, in *display layer options* under the *view* tab, click the drift correction box to view the transformed localizations. Transformed localizations will show up as XC and YC in the exported Insight3 localization software molecule list text file (see Figure 7).
    **Figure 7.**
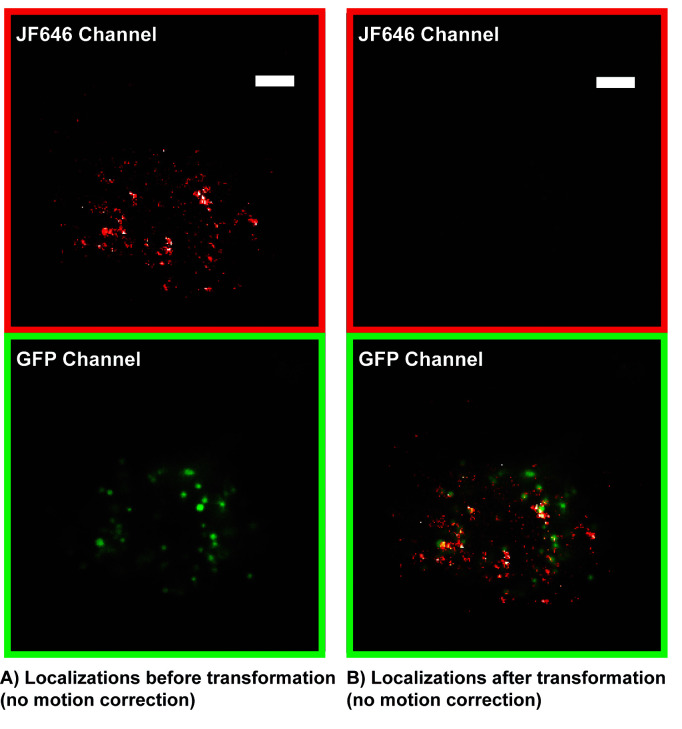
Single molecule localization microscopy (SMLM) localizations (red) before transformation (A) and after transformation (B) superimposed on GFP telomere cluster. The transformation is also used to correct for chromatic aberrations in the optical 4F emission path. Localizations that are not properly transformed cannot be accurately registered with GFP localizations and motion corrected. Apparent incomplete colocalization is due to telomere motion and is corrected during the motion correction analysis. Scale bars: 5 µm.
  4. Calculate a distance matrix between the interpolated GFP localizations for a specific telomere and all single molecule localizations in that frame. If using the provided MATLAB code (motioncorrection.m) this will automatically be done in the cross-correlation section of the motion correction code. The code will loop through all accepted GFP trajectories.
  5. The cross-correlation section of the provided motion correction MATLAB code (motioncorrection.m) identifies single molecule localizations whose distance to a GFP cluster is smaller than the radius of the cluster plus the localization precision of the cluster and the single molecule localizations. The radius of the last GFP localization prior to interpolation should be used as the radius of the interpolated GFP cluster coordinates. The motion correction code (motioncorrection.m) will classify single molecule traces with a minimum of four localizations and with all localizations residing within a GFP cluster as bound and will exclude single molecules traces where some but not all localizations or no localizations reside within a cluster.
  6. To correct for motion of telomeres in PALM images, the motion correction section of provided MATLAB motion correction code (motioncorrection.m) will subtract the coordinates of GFP localizations at a specific frame from the initial GFP localization in the trajectory and apply that subtraction to the single molecule localizations belonging to each GFP cluster and the same frame (see Figure 8).
*Note: A minimum of four bound trajectories with a minimum of four localizations each should be used to obtain enough localizations to be able to motion correct localizations within a telomere trajectory and to calculate downstream metrics such as area and density.*
    **Figure 8.**
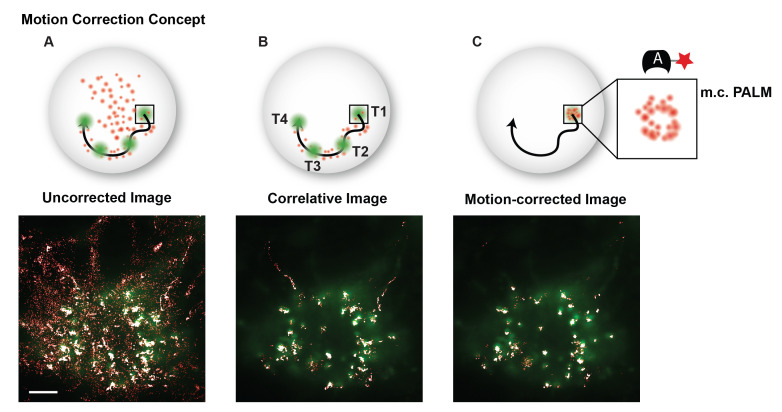
Motion correction example. (A) Superposition of a GFP image and conventional PALM image that includes a majority of freely diffusing and searching fluorescent probes. (B) The correlative conventional and PALM image only depicts PALM localizations that appear in proximity to a GFP cluster at any instance in time and suppresses background from freely diffusing and searching probes. (C) The motion-corrected PALM image super-resolves each moving telomere, which colocalizes with its GFP signal. Scale bar: 5 µm.
  7. Apply a convex hull to the motion-corrected localizations in a cluster using the boundary function in MATLAB (convhull.m) to find the cluster boundary and use this boundary to calculate cluster area.
  8. If calculating localization density, normalize the number of localizations by the cluster duration in the field of view to account for constant photoactivation rate.
  9. Perform desired downstream analysis of structural and dynamic parameters. For instance, correlate cluster density or area with cluster mobility to correlate chromatin structural information with dynamic information. Since single molecule trajectories are assigned to specific GFP telomere trajectories, the diffusion coefficient of the single molecule trajectories can be compared to the mobility of GFP trajectories which provides meaningful information on chromatin rearrangements (see examples in Figure 9). Bulk trace analysis methods such as Gaussian mixture models, Bayesian inference techniques, or displacement analysis techniques can also be applied to trajectories.
  **Figure 9.**
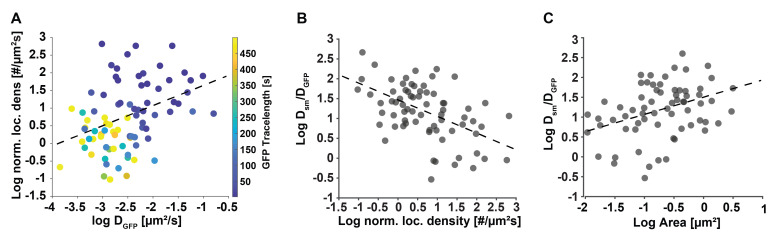
Secondary downstream analysis examples of motion-corrected data. (A) The localization density of individual telomeres has a slight correlation (correlation coefficient = 0.4) with their diffusion coefficient determined with the conventional GFP signal. This result is plausible, since denser, more compacted telomeres can diffuse more freely than less dense and more extended telomeres, whose motion is slowed down. (B) The ratio of average dCas9-MCP single molecule and the telomere diffusion coefficients they reside in presents a metric of relative single molecule re-arrangement and shows a negative correlation (correlation coefficient = -0.52) with the normalized localization density of the telomeres. (C) Likewise, the ratio of average dCas9-MCP diffusion coefficients show a positive correlation (correlation coefficient = 0.48) with the area of the telomeres they reside in. These results are plausible since denser, more compacted telomeres have less ability for relative motion or re-arrangement due to tighter packing compared to less dense, more extended telomeres, which exhibit more relative mobility. Data adapted from Mehra et al. (2022) [17].
